# Immunolabelling perturbs the endogenous and antibody-conjugated elemental concentrations during immuno-mass spectrometry imaging

**DOI:** 10.1007/s00216-023-04967-2

**Published:** 2023-10-06

**Authors:** Monique G. Mello, Mika T. Westerhausen, Thomas E. Lockwood, Prashina Singh, Jonathan Wanagat, David P. Bishop

**Affiliations:** 1 Hyphenated Mass Spectrometry Laboratory, Faculty of Science, University of Technology Sydney, P.O. Box 123, Broadway, NSW 2007, Australia; 2 Division of Geriatrics, Department of Medicine, David Geffen School of Medicine at UCLA, Los Angeles, CA 90095, USA

**Keywords:** LA-ICP-MS imaging, Immuno-mass spectrometry imaging, Endogenous elements

## Abstract

Immuno-mass spectrometry imaging uses lanthanide-conjugated antibodies to spatially quantify biomolecules via laser ablation-inductively coupled plasma-mass spectrometry (LA-ICP-MS). The multi-element capabilities allow for highly multiplexed analyses that may include both conjugated antibodies and endogenous metals to reveal relationships between disease and chemical composition. Sample handling is known to perturb the composition of the endogenous elements, but there has been little investigation into the effects of immunolabelling and coverslipping. Here, we used cryofixed muscle sections to examine the impact of immunolabelling steps on the concentrations of a Gd-conjugated anti-dystrophin primary antibody, and the endogenous metals Cu and Zn. Primary antibody incubation resulted in a decrease in Zn, and an increase in Cu. Zn was removed from the cytoplasm where it was hypothesised to be more labile, whereas concentrated locations of Zn remained in the cell membrane in all samples that underwent the immunostaining process. Cu increased in concentration and was found mostly in the cell membrane. The concentration of the Gd-conjugated antibody when compared to the standard air-dried sample was not significantly different when coverslipped using an organic mounting medium, whereas use of an aqueous mounting medium significantly reduced the concentration of Gd. These results build on the knowledge of how certain sample handling techniques change elemental concentrations and distributions in tissue sections. Immunolabelling steps impact the concentration of endogenous elements, and separate histological sections are required for the quantitative analysis of endogenous elements and biomolecules. Additionally, coverslipping tissue sections for complementary immunohistochemical/immunofluorescent imaging may compromise the integrity of the elemental label, and organic mounting media are recommended over aqueous mounting media.

## Introduction

Elemental bio-imaging (EBI) with laser ablation-inductively coupled plasma-mass spectrometry (LA-ICP-MS) has revealed important relationships between metals and disease. The quantitative spatial imaging has provided insights on the distributions of Cu, Zn, Fe, and Mn in mouse models of Parkinson’s disease [1, 2], and in breast cancer tumours [3]. Immuno-mass spectrometry imaging, or iMSI, was developed to image biomolecule expression by combining EBI with immunolabelling to visualise protein distributions in tissue sections. By using metals as antibody reporters to analyse proteins, biomarkers, and elements, iMSI has revealed biological relationships between metals and proteins, and protein-protein interactions [4-8]. The potential of iMSI has resulted in commercial products including the widely used MaxPar^®^ conjugation kits that conjugate polymers loaded with lanthanide metals to primary antibodies to simultaneously target multiple proteins. The development of these reagents alongside purpose-built LA-ICP-MS systems such as the imaging mass cytometer^™^ has led to its rapid uptake, and highly multiplexed applications are the norm.

Multiplexed analyses have been used to image 32 antigens on breast cancer sections [7], and to simultaneously detect mRNA and proteins [8]. Furthermore, these reagents were used to colocalise the endogenous Fe with tyrosine hydroxylase, a marker for dopamine expression, in murine models of Parkinson’s disease [5]. Metal-conjugated secondary antibodies were used to image tumour biomarkers in breast cancer tissues and study the distribution of β-amyloid peptide with relation to Alzheimer’s disease [4, 9], with both studies also examining the roles of endogenous metals in their samples. While the combination of metal tags and immunolabelling has proven to be a valuable tool, the effects of using such complex techniques on the initial state of the tissues when used in combination with EBI are yet to be fully addressed.

The standard immunolabelling procedure consists of a number of steps which can be categorised into three stages where specimens are prepared, stained, and coverslipped [10, 11]. Each of these steps has the potential to cause disruptions to a sample’s original chemical environment. For quantitative iMSI to realise its potential to supersede qualitative immunohistochemistry (IHC), the basis of many clinical diagnostics, it is of utmost importance that these changes are understood and monitored to ensure accurate, precise, and repeatable analyses. The preservation and preparation of samples for immunolabelling include tissue fixation, embedding, and sectioning. These processes are the most well-studied regarding their impact on elemental concentrations. Formalin fixation is known to leach amino acids, carbohydrates, lipids, phosphates, proteins, and ions including Cl^−^ and K^+^ from the murine brain, alongside a significant increase in the concentrations of Ca, Fe, Cu, and Zn due to a contamination in the formalin solution [12]. Similarly, Hare et al. [13] investigated loss and reuptake of metals during fixation in paraformaldehyde (PFA) and sucrose cryoprotection. They highlighted that metal leaching from fixed and cryoprotected tissue depends on the solubility of the metal involved, particularly group 1 and 2 metals. Additionally, less soluble transition metals are less susceptible to leaching yet still display some degree of loss during fixation and cryoprotection, with Fe, Cu, and Zn decreasing in concentration by approximately 50%. Pushie et al. [14] compared hippocampal Zn distribution in brain hemispheres from mice and rats prepared through either rapid plunge freezing in liquid nitrogen-cooled iso-pentane (RPF, left hemisphere) or through sucrose cryoprotection (SCP, right hemisphere). SCP resulted in substantial Zn redistribution in the hippocampus and was unsuitable for preserving the in vivo hippocampal Zn distribution. Chemical fixation decreased diffusible elements (K, Ca, Na, and Mg) [15], and again cryofixation was shown as the superior method for cell preservation by observing the highest K/Na ratios for cryofixed-freeze dried sections over chemically fixed cells [16]. A comparison of fixatives using Western blot revealed that frozen tissues facilitate recovery of the largest number of proteins from kidney lysates [17]. Meanwhile, Hobrov and Smith used Raman imaging to show chemical fixatives cause a reduction in protein and nucleic acid concentrations when compared to live untreated cells [18]. Fixatives were also seen to cause differences in the colour contrast between cell structures such as the nucleus, collagen fibres, and epithelial tissues upon haematoxylin and eosin staining [19]. Such variabilities increase the chances of discordance between analysts. While other groups have also shown the same effects of chemical fixatives [18-20], there is a lack of information available about the changes occurring during and after the immunolabelling of tissues. This deficit can be attributed to the inability of objectively measuring the changes caused due to the colorimetric detection systems.

The second stage of immunolabelling consists of antigen retrieval, washes, blocks of non-specific binding, cell/tissue permeabilisation, and antibody incubations, depending on the fixative and detection system used [10, 11]. In IHC, specimens are analysed and imaged using a light or fluorescence microscope which typically requires the use of a coverslip. Slides are coverslipped using mounting media to obtain high quality images and are usually left with the coverslip on in the slide box in the event that future analysis is required. There are two main types of mounting media used: organic media such as dibutylphthalate polystyrene xylene (DPX), and aqueous media such as glycerol. The choice of mounting media is dependent on the type of substrate and tags used in the detection system. Most users opt for the organic mounting media that requires additional dehydrations steps; however, an aqueous mount is recommended for fluorescence imaging [21]. Samples stained with metal-conjugated antibodies for iMSI are typically air-dried after the antibody incubation; however, some applications have used multimodal IF/iMSI probes to make use of the higher resolution available with IF imaging to complement quantitative iMSI [7, 22]. Although literature has extensively shown the implications of sample preparation on elemental analytes, the influence of the steps involved in immunolabelling and coverslipping are yet to be completely determined, particularly on the metal conjugated to the antibody.

To address this, we incorporated metal and colorimetric tags to explore the effects of immunolabelling and coverslipping on endogenous elements and the metal-antibody complex. The multimodal detection system consisted of a lanthanide-conjugated primary antibody followed by application of a secondary antibody and an avidin–biotin complex (ABC) with an enzyme tag for conventional IHC staining. This system allowed for high-resolution brightfield microscope imaging of the target biomolecule followed by LA-ICP-MS analysis to detect the endogenous metals and lanthanide-conjugated antibody. LA-ICP-MS analysis is destructive; therefore, samples that are stained with this system must be imaged with a microscope prior to EBI/iMSI. Different immunolabelling steps and mounting mediums were tested on human quadricep muscle biopsies with dystrophin as the model biomolecule, and Zn and Cu as the model endogenous elements as they are known to bind to dystrophin [23]. Dystrophin is a low abundant cytoskeletal protein located in the membrane of the muscle fibre, and it is routinely analysed by IHC or Western blot for the diagnosis of Duchenne muscular dystrophy (DMD) and Becker muscular dystrophy (BMD) which are caused by a decrease or absence of the protein [24].

## Experimental

### Materials

Gadolinium (III) nitrate hexahydrate, copper (II) nitrate hydrate, zinc nitrate hexahydrate, Tris-base, ethylenediaminetetraacetic acid (EDTA), polyethylene glycol (Mn 400), and gelatine from bovine skin (type B) were purchased from Sigma Aldrich (Castle Hill NSW, Australia). Grace Bio-Labs (Bend, OR) supplied 6 Hybriwell^™^ gasket (20 × 9.8 mm) and clear polycarbonate cover with two ports (item number 612107, depth 0.25 mm, volume 50 μL). Ultrapure HNO_3_ and certified Zn, Cu, and Gd standards were supplied by Choice Analytical (Thornleigh, New South Wales, Australia).

A mouse anti-dystrophin antibody (MANDYS8) was purchased from Santa Cruz Biotechnology (Dallas, Texas, USA) and was labelled with the Maxpar^®^ Gd-158 reagent by Fluidigm (South San Francisco, CA, USA). Bloxall blocking solution and a VECTASTAIN^®^ Elite ABC-HRP Kit were purchased from Vector Laboratories (Burlingame, CA, USA), and Tween-20 and 10× TBS were obtained from Bio-Rad (Hercules, CA, USA). Richard-Allan Scientific^™^ Modified Mayer’s Hematoxylin and Richard-Allan Scientific^™^ Cytoseal^™^ were sourced from Thermo Fisher Scientific (Waltham, MA, USA). Aqua-Poly/Mount was purchased from Polysciences (Taipei, Taiwan).

### Matrix-matched gelatine standards

Gelatine standards were prepared using protocols developed by Westerhausen et al. [25]. Ten percent gelatine solutions were prepared by dissolving 100 mg of bovine gelatin at 54 °C for 10 min in 1000 μL of diluent consisting of 10 mM EDTA, 100 mM Tris–HCl (pH 7.4), and 1% w/w PEG400. High concentration salt solutions of gadolinium (III) nitrate hexahydrate, copper (II) nitrate hydrate, and zinc nitrate hexahydrate were used to spike the diluent with desired concentrations of metals prior to being mixed with the aqueous gelatine. Once a homogenous gelatine solution was achieved, the mixture was pipetted onto microscope slides with the use of commercial moulds. The slides were placed into the freezer for at least 30 min until the aliquots of gelatine transformed from transparent to opaque. At this stage, the mould was peeled off and the solid gelatine was left to air-dry and stored at room temperature. Each gelatine mixture was digested using high purity nitric acid (67–70% w/w), diluted 100 times in triplicate, and analysed using solution ICP-MS to determine the exact concentrations of the elements in the produced gel standards. Matrix-matched gelatine standard concentrations of Cu, Zn, and Gd are provided in Supplementary Table 1.

### Instrumentation

All analyses were performed on a New Wave Research 193 nm ArF excimer laser coupled to an Agilent 7700cs ICP-MS. Argon was used as the carrier gas and hydrogen was used as a collision gas to mitigate polyatomic interferences on Cu and Zn. The optimal ICP-MS and laser conditions are summarised in Table 1. Laser ablation and ICP-MS integration parameters were set according to the equation described by Lear et al. [26]. A 15 μm spot size was used to raster across samples at a rate of 60 μm s^−1^ with a pulse rate of 20 Hz, with Cu^63^, Zn^66^, and Gd^156^ monitored with a total ICP-MS integration time of 0.25 s.

### Immunolabelling

Human muscle biopsies were obtained with informed consent from adult subjects without muscle disease at the Center for Duchenne Muscular Dystrophy at the University of California Los Angeles under an IRB-approved protocol (#11-001087). Skeletal muscle biopsies from the vastus lateralis were embedded in OCT, frozen in liquid nitrogen, sectioned at 10 μm, and stored at −80 °C.

Samples were prepared in triplicate for each of the sample preparation methods. To obtain baseline values of the endogenous elements, cryosections were washed with distilled water and air-dried before detection by LA-ICP-MS (see Table 2, sample A). The immunolabelling protocol of the human muscle biopsy cryosections was as follows: the samples were air-dried for 30 min, washed twice with 1×TBS, and incubated with Bloxall blocking reagent for 10 min. After blocking, the sections were washed with TBS-T (0.1% Tween-20) before a 30 min incubation with gadolinium-labelled primary anti-dystrophin antibody (1:500 dilution). The slides were washed with TBS, rinsed with double distilled H_2_O, and allowed to air-dry overnight (sample B).

Immunolabelling of the samples prepared for multimodal detection followed the same protocol as above until the antibody incubation. After the primary antibody incubation, slides were washed with 1 × TBS and the VECTASTAIN^®^ Elite ABC-HRP Kit was used as per manufacturer’s instructions. The slides were rinsed with distilled water once the ImmPACT DAB chromogen had developed colour. At this point, sections were either washed and air-dried (C), cover-slipped with Aqua-Poly/Mount (aqueous mounting media) (D), dehydrated with a series of ethanol washes and cover-slipped with Cytoseal^™^ (organic mounting media) (E), or dehydrated, counterstained with Richard-Allan Scientific^™^ Modified Mayer’s Hematoxylin, and coverslipped with Cytoseal^™^ (F).

The coverslips of samples D-F were removed prior to LA-ICP-MS analysis. Slides with aqueous mounts were left in distilled water until the coverslips could be easily removed without causing any damage to the tissue samples. Samples were then air-dried after additional water washes. Two xylene washes were used to remove the organic mounted coverslips and mounting media. Care was taken to ensure that all 18 samples were ablated in the same region of the serial sections.

### Image processing and statistical analysis

LA-ICP-MS data was spatially resolved and quantified using external calibration on an in-house developed software (Pew^2^) [27] prior to being processed in MATLAB. 3-Level k-means and 2-level k-means segmentation were performed together according to our previous method to discriminate between positive signal of Gd in the cell membrane and the null signal in the sarcoplasm [28]. Descriptive statistics were obtained for all the processed images using MATLAB which were then analysed for significance using two-tailed *T*-tests in Origin Labs software. The statistical analysis was completed at a significance level of *α* = 0.05 unless stated otherwise, and all samples were processed and analysed using the same steps to ensure a fair comparison between different treatments. The images were produced from quantified raw data with the application of a bicubic filter for the smoothing of square pixels.

## Results and discussion

### Analytical figures of merit

The limits of detection (LODs) of Cu, Zn, and Gd were calculated by obtaining the concentrations corresponding to three times the signal-to-noise, and are 6, 2, and 0.004 μg kg^−1^, respectively. All calibration curves yielded linearity of more than 0.999 (for the concentration range, see Supplementary Table 1).

### Influence of immunolabelling on the elements

**Fig. 1:**
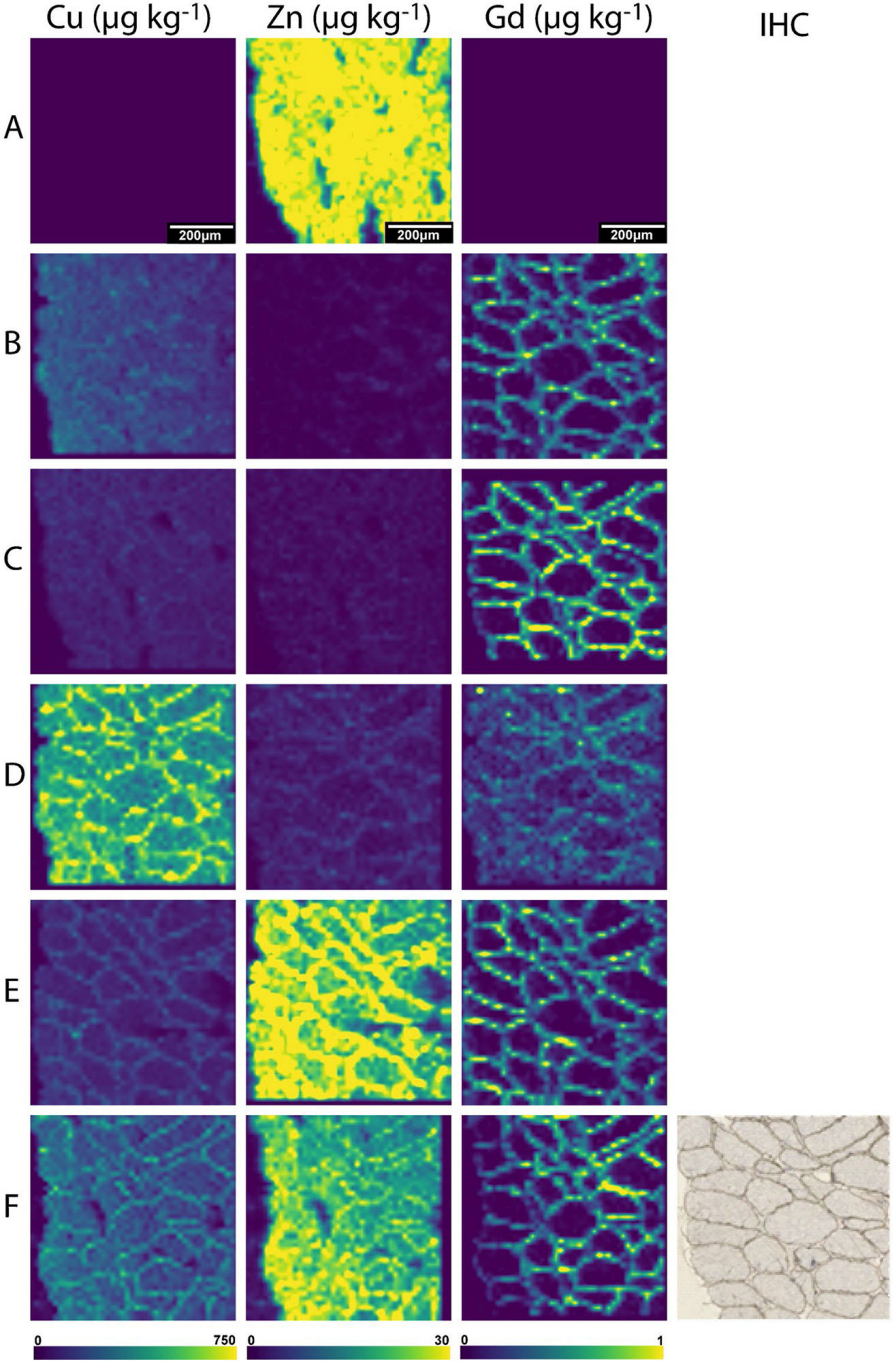
Representative images of sections depicting Cu, Zn, and Gd distributions with no immunostaining for a baseline (**A**), post-application of primary antibody (**B**), primary and secondary antibody air-dried (**C**), coverslipped with an aqueous mount (**D**), coverslipped with an organic mount (**E**), or counterstained before being coverslipped with an organic mount (**F**). A bicubic interpolation has been applied to these images with the scale bar representing 200 μm. The light microscope image of the sample was obtained with multimodal detection (optical and elemental) on the same section (**F**), where the brown precipitate corresponds to the protein of interest (dystrophin)

The concentrations of Cu and Zn obtained from consecutive histological sections that underwent the differing sample preparation protocols are summarised in Table 3. The concentration of Cu in the control sample (A) was below the instrument quantification limit, and Zn was approximately 30 μg kg^−1^ with broad uniform distribution across the section (see Fig. 1). These values were lower than those reported by Sewry et al. who found concentrations of 0.17 and 2.5 μmol g^−1^ (10.8 and 163.5 mg kg^−1^) for Cu and Zn respectively in human muscle tissue using X-ray fluorescence spectroscopy [29]. This is an older study which used a single point calibration from a Cr spike onto the sample. A recent study used LA-ICP-MS and dried microdroplets onto filter paper as calibration standards to analyse mouse cryosections to find 1.43 mg kg^−1^ of Cu and 23.9 mg kg^−1^ of Zn present in muscle tissue [30]. The values reported in the literature and this study were different. This may be due to the different sample types (mouse vs human) or the method of standard preparation and instrumentation used. However, these techniques tend to produce relative quantitative results, and the trend of low Cu levels and high Zn content observed in this study was consistent.

The adverse effects of the immunolabelling protocols on labile metals are shown in Table 3 and exemplified in Fig. 1. The Zn distribution changed from uniform (A) to localised in the cell membrane (D–F). A large change was observed in the concentration of Cu, with distribution broadly in the cell membrane. This is consistent with the findings of Roudeau et al. who used staining protocols that comprised of cryofixing, permeabilisation, and antibody (1° and 2°) incubations and employed synchrotron X-ray chemical imaging to qualitatively show the redistribution and loss of P, S, K, Mn, Fe, and Zn after undergoing immunofluorescence staining [31]. In contrast, Matsuyama et al. revealed that diffusible elements (K, Ca, Na, and Mg) decreased upon chemical fixing while elements presumed to be tightly bound to proteins (Zn and Cu) remained unaffected [15]. This explains the retention of low levels of Zn in the cell membrane in B–D, where it is known to bind to muscle proteins such as dystrophin [23]. Perrin et al. also reported a loss of labile metals in single cells from chemical fixatives and washing buffers [16]. Hachmöller et al. [32] investigated the influence of immunostaining on the Cu content of liver samples, with LA-ICP-MS and rhodamine staining used to visualise the distribution of Cu. Sections stained with rhodamine showed altered copper distribution and significantly decreased copper concentrations compared to unstained or conventionally (H&E) stained sections. Rhodamine binds to copper to form a copper rhodamine complex that is soluble and possibly removed from the sample during the staining process. Hackett et al. [12] reported that SCP during sample preparation contributed to Zn redistribution over cell membranes and its binding beyond the tissue, principally tissues containing large proportions of labile and mobile metal ions. They found both leached and enriched Zn levels depending on the SCP tissue region analysed. Similarly, Hare et al. [13] noticed that Zn and Cu significantly leached during the fixation step in SCP samples. The results obtained here support the notion that metals are mobilised from specific areas to others depending on their state during sample preparation.

**Fig. 2:**
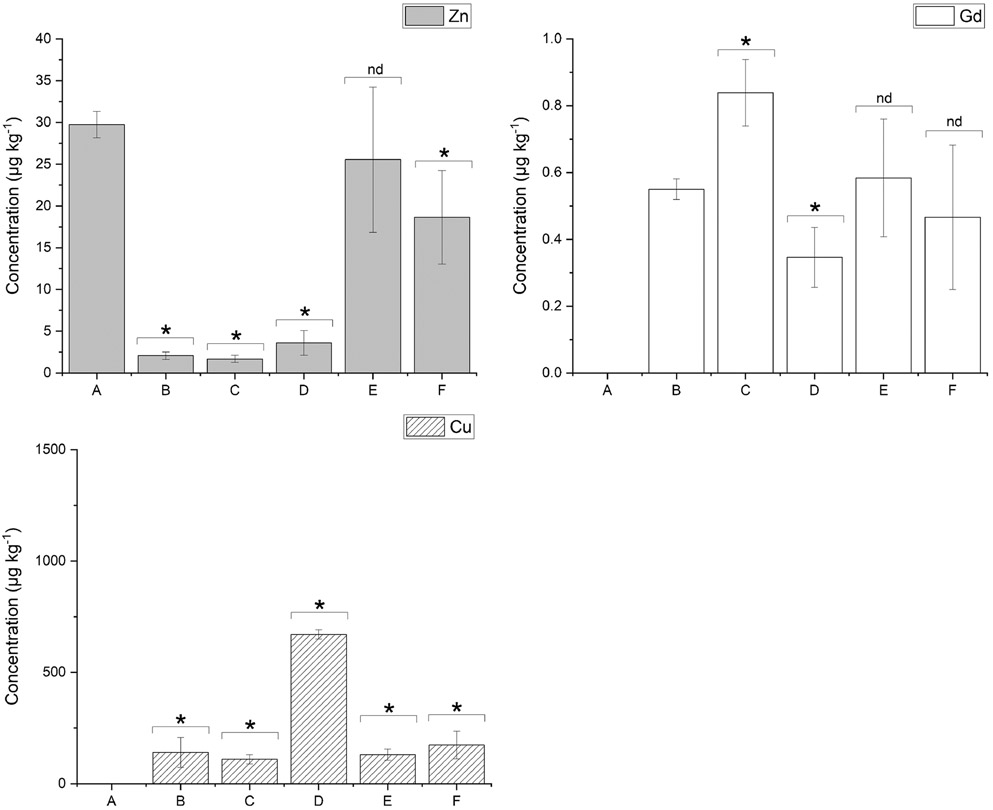
Concentrations and results of the two-tailed *T*-tests of Cu, Zn, and Gd for all sample preparation protocols. * denotes significant difference between protocols (*p* = 0.05), and nd denotes no significant difference

The multimodal detection method used an additional secondary antibody and DAB precipitate for standard IHC visualisation in addition to LA-ICP-MS imaging. This is beneficial as it allows confirmation of the LA-ICP-MS images against higher resolution microscope images on the same section (Fig. 1F). The average concentrations of Gd across the images were processed after k-means segmentation [28] (see Table 3), and a two-tailed *T*-test was used to compare the effects of the additional secondary antibody and the DAB precipitate on the Gd concentrations and hence the primary antibody (Fig. 2, Supplementary Table 2). The *T*-test revealed a significant increase in Gd concentration for the air-dried samples stained with a secondary antibody (C). The DAB precipitate provided a hydrophobic mask that prohibits interactions which may result in losses of the metal tag or the antibody in immunolabelling steps such as the TBS and water washes which occurred after the antibody incubation in sample (B). Von Schoenfeld et al. recently reported that the application of a secondary antibody and the DAB chromogen protected the metal label on primary antibodies [33]. They were initially unable to detect a metal-conjugated primary antibody by imaging mass cytometry; however, application of a fluorophore-conjugated secondary antibody detected the metal-conjugated primary antibody. This indicated that it was the metal or polymer being lost during their immunolabelling steps and not the primary antibody. While the authors could not find other studies reporting on changes to the primary antibody during immunolabelling, Tangrea et al. [34] investigated the effects of IHC on the quality and recoveries of DNA, RNA, and protein in numerous types of tissues specimens. They showed that DNA and most of the proteins tested were not damaged during the immunolabelling procedure by using liquid chromatography-mass spectrometry to reveal a difference of less than 10% between stained and unstained samples. This provides further evidence that the increased quantity of Gd in sample (C) compared to sample (B) (Fig. 1) was most likely related to the metal conjugate or to the antibody-antigen interaction, as opposed to dystrophin itself being impacted. As such, these results promote the use of a secondary antibody as it preserves the primary antibody-antigen bond during immunostaining [35, 36].

### Changes in tissue composition due to coverslipping mounts and counterstain

Here, we investigated the effects of coverslipping with aqueous or organic mounting media by comparing the concentrations of Cu, Zn, and Gd in samples that were either air-dried or coverslipped post multimodal immunolabelling. To establish baseline Cu, Zn, and Gd levels prior to coverslipping, samples were immunolabelled and air-dried only. Moreover, a set of samples were counterstained with haematoxylin prior to coverslipping to investigate its influence on the analytes. The resultant images and corresponding concentrations with standard deviation showing the distributions of Cu, Zn, and Gd from LA-ICP-MS analysis of the samples can be found in Fig. 1 and Table 3, respectively. The samples that were coverslipped show an altered distribution of Cu and Zn mainly located in the cell membrane (see Fig. 1D-F compared to A). As expected, the distribution of the immunolabel using Gd as the proxy analyte did not change; however, (D) is not as clear as the remaining images. Two-tailed *T*-tests were used to examine if the Cu, Zn, and Gd concentrations were significantly affected by the different post staining treatments (*α* = 0.05). Figure 2 shows the mean and standard deviation of Cu, Zn, and Gd for all treatments, as well as the population comparison between treatments.

The two-tailed *T*-tests revealed a significant difference between the baseline Cu and Zn concentrations (A) when compared to the concentrations obtained from samples (B–F), with the exception of Zn in (E). Cu was below LOQ in the baseline sample (A), and exhibited an increase of concentration of at least 100 × in all samples after immunolabelling. While changes in the percent recovery of endogenous metals during immunolabelling was recently described elsewhere [37], the results presented here also indicated that the type of coverslipping technique used had a large effect on the analyte. The Cu content in samples that were coverslipped with an aqueous mounting media (D) had almost five times the amount of Cu than the air-dried samples (B and C). Though an increase in Cu concentration was also seen in the other samples from the immunolabelling procedure, the organic mount and counterstain did not greatly affect the Cu content in the tissues over the air-dried immunolabelled sample B. Since only the aqueous mounted samples (D) had a large influx of Cu over (B), it was postulated that the extra Cu contamination was directly related to the mounting media. Counterstaining with haematoxylin (F) did not affect the distribution and concentration of Cu compared to (E), which agreed with an investigation into the effects of immunolabelling by Hachmoller et al. [32]. The Zn contents in the immunolabelled and air-dried (B and C) and aqueous coverslipped sections (D) were significantly lower with concentrations approximately ten times less than the baseline sample (A). Samples (E) and (F), which were coverslipped with an organic mount, did not show the same decrease in concentration, with (E) not significantly different to (A). These differences suggest that the steps involved in coverslipping with an organic mount, such as dehydration or the mounting media itself, contributed to the increase in Zn. The counterstaining did not increase the Zn concentration as samples (E) and (F) were within the standard deviation of each other. Levels of Zn and Cu in the solvents and reagents were investigated to understand each element’s potential contributions during the IHC preparation steps. Trace concentrations of Zn were found in both ethanol and xylene but would be insufficient to cause the observed contamination. The mouse-on-mouse blocking reagent tested contained high levels of Cu and Zn (Supplementary Table 3) and may represent a source of contamination [37].

It was expected that the extra sample handling steps would impact the endogenous elements; however, the changes on the Gd reporter were surprising. The results revealed that application of the secondary alone produced a significant increase in concentration of Gd (C) from the baseline sample (B), and that the use of an aqueous mounting media significantly decreased the concentration (D). The samples that had an organic mounting media applied (E and F) did not exhibit significant changes in the Gd concentration. The significant decrease in Gd in sample (D) was hypothesised to be due to two factors: competition for the DTPA binding site in the Maxpar polymer, and changes to the epitope during rehydration as the coverslips were removed, affecting the antibody-antigen interaction which resulted in a reduction of binding. DTPA, although a strong chelating agent of Gd, can be displaced by other metal ions in solution such as Cu, Zn, Mg, and Ca when they are in excess compared to Gd [38]. The results shown in Table 3 and Fig. 1 show a 670 × increase of Cu in sample (D), providing evidence that a large excess was available for competition with the Gd. Changes in the water content of a sample affect the three-dimensional conformation of a protein [39] and therefore the availability of the epitope through either direct changes to the epitope or to the neighbouring proteins that play a role in epitope availability [40]. Furthermore, changes of sample pH due to the mounting media may have also played a role as it is known to disrupt the antigen–antibody binding equilibrium [41].

Aqueous mounts are usually used for fluorescent imaging to obtain high-resolution images. Complementary immunofluorescent imaging has been conducted prior to imaging mass cytometry [7, 8] to validate the antibodies used. While it is not clear whether the changes in the reporter tag identified here are occurring during the application of the mount or the removal of the coverslip, users of aqueous mounts need to be aware of these changes occurring as it can misrepresent the quantity and distribution of target biomolecules in the sample.

Samples that were dehydrated prior to being coverslipped with an organic mounting media showed no significant difference in mean Gd concentration from the baseline values. While the primary purpose of tissue dehydration is to remove water from the sections to promote miscibility of the tissue and the organic mounting media, it also inhibits solubility of molecules and stops biomolecule mobility. The lack of statistical change in Gd concentration of these samples (E and F) indicates that antibodies are less likely to be removed during coverslipping or removing the coverslip if the tissues are in the dehydrated state. Additionally, while there was a decrease in Gd concentration in samples that had been counterstained (F), no significant difference in Gd concentrations was detected when compared to no counterstaining (E). This suggests that the primary antibody and antigen interaction is not disrupted from additional staining steps like it is from exposure to aqueous mounting substances.

The results obtained here confirm that a DAB precipitate provides protection for the metal label [33], with sample (C) showing the highest concentration of Gd. However, if the work requires high-resolution microscope images, then organic coverslipping should be considered over water-based substances. Use of an organic mounting media is also considered best practice for microscopists so no large change to existing workflows is required [42] if there is a widespread uptake of iMSI. As microscopy will always be an upstream analysis to EBI/iMSI, newer methods of mounting should also be considered for future investigation such as hydrogel [43] and automated coverslipping methods [44].

A number of applications have looked at transition metals simultaneously to an immunolabelled probe without investigating the impact of the immunolabelling procedures on the endogenous elements. Hare et al. [5] found Fe colocalised with tyrosine, a marker for dopamine, in a Parkinson’s mouse model, and Paul et al. [45] investigated the spatial distribution of Fe, Cu, and Zn alongside an anti-tyrosine hydroxylase marker in murine brains. Cruz-Alonso et al. [46] imaged Cu and Zn and aimed to correlate them with metallothionein; however, no correlation was observed. The same authors imaged ferroportin and Fe in hippocampal sections from AD brain samples and compared against healthy control brains, and while Fe increased in the AD sections, no clear correlation was seen between Fe and ferroportin [47]. The lack of correlation in the latter two cases could be a result of changes to the endogenous metal concentration and distribution during the immunolabelling process we identified here. The differences in these studies highlight that the redistribution of the elements may be specific to the tissue type, the element, and the immunolabelling protocols used.

A limitation in the current study is that only the endogenous Cu and Zn content and distribution were examined. Other trace endogenous elements that exhibit distinct binding behaviours in proteins such as Fe and Mn may be impacted differently. Recently, Schaier et al. [37] undertook a similar study to that presented here, looking at the impact of immunolabelling on the endogenous elements in tumour sections, without investigating secondary visualisation, coverslipping, or changes to the elemental concentration of the metal-conjugated antibody. Their results for Cu and Zn confirm those outlined above, with a change in distribution and a large loss of Zn and an increase of Cu observed across the tissue sections during immunolabelling. Again, similar to our results, high Cu concentrations were observed in blocking reagents, which may be the source in the tissues. They did extend their search, and found that while the concentration of Fe changed, its distribution did not, and it could therefore be qualitatively assessed alongside the immunolabelled biomolecules.

The addition of coverslips, while not necessary for iMSI, is part of standard immunolabelling protocols in clinical pathology and biological research laboratories. It is also essential when samples are transported between laboratories for analysis and cross validation to ensure the integrity of the sample is maintained. When developing methods that have broader applicability outside the specialised research field, it is important that we do not deviate from these standard protocols to ensure ease of uptake. Overall, the differences in the endogenous metals analysed seem to be heavily influenced by the type of mounting media used. Hence, the mounting mediums would have to be analysed by solution ICP-MS to confirm if they were the source of contamination. The findings here further reiterate the use of consecutive sections for studies investigating colocalisation to have an accurate representation of metals in biological samples.

## Conclusion

The results obtained here show that care must be taken when concurrently investigating endogenous elements alongside immunolabelled biomolecules. In the tissues we investigated, the concentrations and distributions of Zn and Cu were significantly altered during the immunolabelling steps. Additionally, the process of adding a secondary antibody and visualising with DAB significantly altered the concentration of the metal conjugate, as did the process of coverslipping with an aqueous mounting media. The observed changes in the endogenous metals cannot be extrapolated to other tissue types; however, those observed on the metal conjugate during mounting and coverslipping are more likely to be independent of the sample. Future studies investigating endogenous elements alongside a metal-conjugated immunolabel should be assessed to avoid misinterpretation of the results.

## Supplementary Material

Supplementary file1

## Figures and Tables

**Table 1:** ICP-MS and laser conditions used for analysis of muscle tissues

	Conditions
Laser ablation	
Wavelength	193 nm
Laser power	5%
Laser power density (energy)	0.3 mJ/cm^2^
Laser bean diameter	15 μm
Scan speed	60 μm s^−1^
Frequency	20 Hz
ICP-MS	
RF power source	1350 W
Carrier gas flow	1.05 L min^−1^
Sample depth	4 mm
Extract lens 1 and 2	4.5 V, −125 V
Omega bias, lens	−80 V, 13.2 V
Octopole RF	180 V
Octopole bias	−18 V
Collision gas	H_2_, 3.1 mL min^−1^

**Table 2:** Summary of the sample preparation steps performed; each treatment was completed in triplicate resulted in a total of 18 samples

Sample	Air-dried	Primary antibody	Secondary antibody	Counterstain	Aqueous coverslip	Organic coverslip	Coverslip removed
A	x					
B	x	x				
C	x	x	x			
D		x	x		x		x
E		x	x			x	x
F		x	x	x		x	x

**Table 3:** The concentrations for Cu, Zn, and Gd obtained with the different sample preparation protocols

Sample ID	Cu μg kg^−1^)	Zn μg kg^−1^)	Gd μg kg^−1^)
A	< LOQ	29.7 ± 1.6	< LOD
B	140.8 ± 67.4	< LOQ	0.55 ± 0.03
C	109.7 ± 20.7	< LOQ	0.84 ± 0.10
D	670.4 ± 20.5	< LOQ	0.35 ± 0.09
E	130.3 ± 25.1	25.6 ± 8.7	0.58 ± 0.18
F	173.8 ± 62.0	18.6 ± 5.6	0.47 ± 0.22
