# Supplementary material for: Immunolabelling perturbs the endogenous and antibody-conjugated elemental concentrations during immuno-mass spectrometry imaging
Source: Anal Bioanal Chem. Author manuscript; Available in PMC 2025 May 1. (PMC10997740; doi:10.1007/s00216-023-04967-2)
Supplement: Supplementary file1 [file NIHMS1943403-supplement-Supplementary_file1.docx]

**Supplementary**

Supplementary Table 1: Matrix-matched gelatine standard concentrations of Cu, Zn and Gd.

|  | Concentration (µg kg^-1^) | | |
| --- | --- | --- | --- |
| Level | **Cu** | **Zn** | **Gd** |
| Blank | 0 | 0 | 0 |
| 1 | 24.4 | 1.9 | 0.2 |
| 2 | 129.6 | 6.8 | 1.9 |
| 3 | 655.6 | 31.4 | 10.2 |
| 4 | 1300.3 | 60.2 | 20.2 |
| 5 | 2604.8 | 123.4 | 41.4 |

Supplementary Table 2: Two tailed t-Test.

| Label (target) | Treatment | Concentration (μg kg^−1^) | %RSD | *t^a*^* |
| --- | --- | --- | --- | --- |
| Cu | **A** | 0.82 | 8.91 | -- |
|  | B | 140.8 | 47.9 | 3.598 |
|  | C | 109.7 | 18.9 | 9.087 |
|  | D | 670.4 | 3.1 | 56.516 |
|  | E | 130.3 | 19.3 | 8.928 |
|  | F | 173.8 | 35.7 | 4.830 |
| Zn | **A** | 29.74 | 5.35 | -- |
|  | B | 2.1 | 21.0 | 29.053 |
|  | C | 1.7 | 25.2 | 23.259 |
|  | D | 3.6 | 41.2 | 20.851 |
|  | E | 25.6 | 34.1 | 0.819 |
|  | F | 18.6 | 30.0 | 3.304 |
| Gd | A | -- | -- | -- |
|  | **B** | 0.55 | 5.57 | -- |
|  | C | 0.84 | 11.86 | 4.805 |
|  | D | 0.35 | 25.96 | 3.718 |
|  | E | 0.58 | 30.16 | 0.328 |
|  | F | 0.47 | 46.40 | 0.667 |

*t^a^* < critical value t = 2.306 for two-tailed test (*p* = 0.05), the difference of the population mean is not significantly different from the test difference.

*All Treatments results follow a normal distribution.

Supplementary Table 3: Concentrations of Cu, Zn and Gd found in ethanol, xylene, and mouse-on mouse (MOM) blocking reagent.

| Solvents | Cu (µg kg^-1^) | Zn (µg kg^-1^) | Gd (µg kg^-1^) |
| --- | --- | --- | --- |
| Ethanol | <LOD | 3.82 ± 0.86 | <LOD |
| Xylene | <LOD | 5.05 ± 5.06 | <LOD |
| MOM | 873.06 | 962.62 | <LOD |
